# Effectiveness of Data Augmentation for Localization in WSNs Using Deep Learning for the Internet of Things

**DOI:** 10.3390/s24020430

**Published:** 2024-01-10

**Authors:** Jehan Esheh, Sofiene Affes

**Affiliations:** EMT Centre (Energy, Materials and Telecommunications), INRS (Institut National de la Recherche Scientifique), Université du Québec, Montréal, QC H5A 1K6, Canada

**Keywords:** range-free localization, neural networks, data augmentation, wireless sensor networks

## Abstract

Wireless sensor networks (WSNs) have become widely popular and are extensively used for various sensor communication applications due to their flexibility and cost effectiveness, especially for applications where localization is a main challenge. Furthermore, the Dv-hop algorithm is a range-free localization algorithm commonly used in WSNs. Despite its simplicity and low hardware requirements, it does suffer from limitations in terms of localization accuracy. In this article, we develop an accurate Deep Learning (DL)-based range-free localization for WSN applications in the Internet of things (IoT). To improve the localization performance, we exploit a deep neural network (DNN) to correct the estimated distance between the unknown nodes (i.e., position-unaware) and the anchor nodes (i.e., position-aware) without burdening the IoT cost. DL needs large training data to yield accurate results, and the DNN is no stranger. The efficacy of machine learning, including DNNs, hinges on access to substantial training data for optimal performance. However, to address this challenge, we propose a solution through the implementation of a Data Augmentation Strategy (DAS). This strategy involves the strategic creation of multiple virtual anchors around the existing real anchors. Consequently, this process generates more training data and significantly increases data size. We prove that DAS can provide the DNNs with sufficient training data, and ultimately making it more feasible for WSNs and the IoT to fully benefit from low-cost DNN-aided localization. The simulation results indicate that the accuracy of the proposed (Dv-hop with DNN correction) surpasses that of Dv-hop.

## 1. Introduction

In recent decades, with the advancements in IoT technologies, the intelligent perception and management of objects have become achievable through the connection of things and people [1]. WSNs have played an increasingly significant role in the IoT by facilitating the real-time sensing, collecting, and processing of information. The inherent characteristics of node location make it an essential prerequisite for many functions. During the last decade, this topic has motivated extensive research endeavors that have resulted in several interesting localization algorithms [2]. As the demand for location-based services continues to grow, the accuracy of node localization significantly impacts various application areas, such as city surveillance, and smart homes [3,4]. So far, many localization algorithms have been proposed in the literature and mainly fall into two categories: range-based and range-free algorithms. Range-based localization utilizes the measurements of the received signal attributes such as angle of arriving signal (AOA) [5], received signal strength (RSS) [6], and time of arrival (TOA) [7]. While range-based algorithms are known for their high accuracy, they are impractical for WSNs due to their high-power requirements for communication between anchors and regular nodes, especially in the case of small battery-powered units. Furthermore, these algorithms are susceptible to interference and fading, often necessitating additional hardware, thereby burdening both the WSN and the IoT costs. Range-free localization techniques are variations of the well-known Dv-hop algorithm that simply converts the numbers of hops into coordinates [8,9,10]. Such techniques do not require any additional hardware, in contrast to range-based methods, but are relatively much less accurate. To improve localization performance, researchers have recently resorted to machine learning (ML) or DL. Several ML techniques have been investigated in this context such as, namely, support vector machine (SVM), artificial neural network (ANN), etc. [11,12,13,14]. The key hurdle these techniques face in common is the requirement of large training data sets. The larger they are, the more accurate they will be in correcting the estimated node positions However, such data, generated very often by anchors deployed in few numbers due to the expensive GPS technology they integrate, are relatively scarce, thereby limiting any potential ML-driven performance improvement. Therefore, to improve localization accuracy, we develop a precise, and cost-efficient DNN-based range-free localization approach for WSN applications in the IoT. To tackle the issue of limited data for training the proposed DNN, we are also working on an efficient DAS.

The rest of the paper is organized as follows. Section 2 introduces the range-free localization process. Section 3 describes the implementation of data augmentation in WSN application. The architecture processing of the DNN is described in Section 4. In Section 5, several experiments are performed, and the experimental results are analyzed. Finally, in Section 6 we conclude the paper with a summary.

## 2. Localization Process

**Figure 1:**
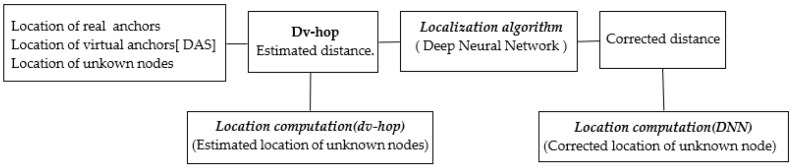
Overview of localization process.

The aim of sensor localization is to determine the locations of unknown nodes. The localization process identifies the positions of these unknown nodes based on input data. In our case, the input data consists of the locations of both real anchors and their virtual counterparts, along with the unknown node. An overview of the localization process is depicted in Figure 1.

This section explains the estimated distance and location computation process, while the remaining steps will be covered in the next section.

For estimation of the distance between anchor and unknown nodes, we assume each node communicates with an anchor node through a multi-hop path by using a localization algorithm [10]. Firstly, all unknown nodes in the network obtain minimal hop counts to every anchor node. During the second phase, when an anchor node obtains hop counts to other anchors, it calculates an average distance for one hop, which is subsequently disseminated to the entire network. Anchor node i estimates the average hop size using the following equation:

(1) $${\mathrm{Hopsize}}_{i}=\frac{\sum_{i\neq j}\sqrt{{\left(x_{i}-x_{j}\right)}^{2}+{\left(y_{i}-y_{j}\right)}^{2}}}{\sum_{\mathrm{ij}\neq h_{\mathrm{ij}}}}\;,$$

where $\left(x_{i},y_{i}\right)$ and $\left(x_{j},y_{j}\right)$ are the known coordinates of anchors i and j, respectively, and $h_{\mathrm{ij}}$ is the minimum number of hops between them

Upon receiving the hop size from the anchor nodes with the least hops between them, every unknown node computes its distance $\left(d_{i}\right)$ to each anchor node I using the hop size denoted earlier in Equation (1) as ${\mathrm{Hopsize}}_{I}$ and the minimum hop count denoted here as hops as follows:

(2) $$d_{i}=\mathrm{hops}\times {\mathrm{Hopsize}}_{i}.$$

Hence, the location of the unknown node can be estimated by solving the following set of equations:

(3) $$\left\{\begin{array}{c} {\left(\hat{x}-x_{1}\right)}^{2}+{\left(\hat{y}-y_{1}\right)}^{2}=d_{1}^{2}\\ {\left(\hat{x}-x_{2}\right)}^{2}+{\left(\hat{y}-y_{2}\right)}^{2}=d_{2}^{2}\\ \vdots \\ {\left(\hat{x}-x_{n}\right)}^{2}+{\left(\hat{y}-y_{n}\right)}^{2}=d_{n}^{2} \end{array}\right.;$$

where $\left(x_{i},y_{i}\right)$ denote the coordinates of anchor i = 1, …, n and $\left(\hat{x},\hat{y}\right)$ are the coordinates of the unknown node. Indeed, Equation (3) could be linearized and solved under the minimum mean square error (MMSE) criterion to estimate the coordinates of the unknown node $\left(\hat{x},\hat{y}\right)$ as follows:

(4) $$\left[\begin{array}{c} \hat{x}\\ \hat{y} \end{array}\right]=\frac{-1}{2}{\left(\psi^{T}\psi \right)}^{-1}\psi^{T}\varphi ,$$

where:

(5) $$\psi =\left[\begin{array}{cc} x_{1}-x_{n} & y_{1}-y_{n}\\ \begin{array}{c} x_{2}-x_{n}\\ \vdots \end{array} & \begin{array}{c} y_{2}-y_{n}\\ \vdots \end{array}\\ x_{n-1}-x_{n} & y_{n-1}-y_{n} \end{array}\right]\;,$$

and

(6) $$\varphi =\left[\begin{array}{c} d_{1}^{2}-d_{n}^{2}-x_{1}^{2}+x_{n}^{2}-y_{1}^{2}+y_{n}^{2}\\ d_{2}^{2}-d_{n}^{2}-x_{2}^{2}+x_{n}^{2}-y_{2}^{2}+y_{n}^{2}\\ \begin{array}{c} \vdots \\ d_{n-1}^{2}-d_{n}^{2}-x_{n-1}^{2}+x_{n}^{2}-y_{n-1}^{2}+y_{n}^{2} \end{array} \end{array}\right]\;.$$

## 3. Data Augmentation in WSN Application

Typical augmentation techniques applied to images involve a range of transformations such as translation, blurring, flipping, rotation, and the introduction of various types of noise to data samples. These techniques are well established in the field, and diverse DASs are tailored to specific problems. For example, in the context of the MNIST database of handwritten digits, researchers have explored augmentation techniques [15]. In the field of machine learning, especially for researchers working with techniques like Generative Adversarial Networks (GANs), the limited availability of large datasets for effective training poses a significant challenge. In response to this challenge, a novel concept known as “virtual big data” is introduced [16]. This concept involves the generation of synthetic or virtual datasets that mimic the characteristics of real-world data, offering a solution in situations where obtaining extensive real-world data is impractical. In consideration of the small datasets of chemical reactions, the data-driven model suffers from the difficulty of low accuracy in the prediction tasks of chemical reactions. To tackle this, the model integrated with the strategies of data augmentation [17]. The data augmentation is used to improve the performance of data-driven reaction prediction models by increasing the sample size using fake data augmentation [18].

**Figure 2:**
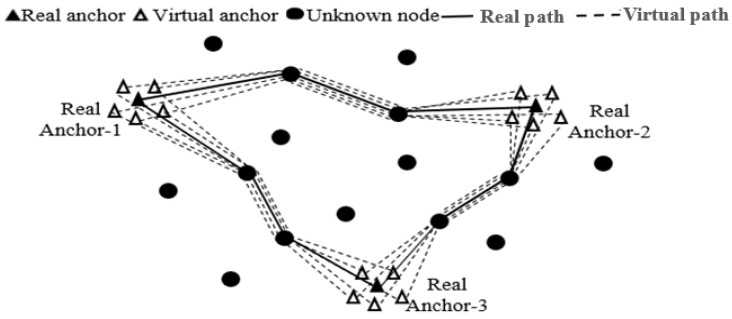
Creation of virtual anchors.

In what follows, we will employ a similar approach by utilizing data augmentation to increase the dataset. This involves creating multiple copies of virtual anchors for each real anchor around its position, as illustrated in Figure 2. The coordinates of these new virtual anchors are mathematically represented by Equation (7). This data augmentation technique is applied to enhance the dataset, generating additional instances of virtual anchors to increase the training data.

As shown in Figure 2, there are three anchors (A = 3), and each anchor is surrounded by five virtual anchors (V = 5). The coordinates $\left({\tilde{x}}_{k},{\tilde{y}}_{k}\right)$ of the virtual anchor near the real anchor i can be obtained by adding a span multiplied by a random Gaussian variation ( $\Delta x_{k},\;\Delta y_{k}$ ), denoted as (Vcx, Vcy), as depicted in Algorithm 1 (steps 6 and 7), to the coordinates of the anchor i ( $x_{i},y_{i})$ for (i = 1, …, A) and (k = 1, …, V) as follows:

(7) $$\left\{\begin{array}{c} \left({\tilde{x}}_{1},{\tilde{y}}_{1}\right)=\left(x_{i}\pm \Delta x_{1},x_{i}\pm \Delta y_{1}\right)\\ \left({\tilde{x}}_{2},{\tilde{y}}_{2}\right)=\left(x_{i}\pm \Delta x_{2},x_{i}\pm \Delta y_{2}\right)\\ \begin{array}{c} \vdots \\ \left({\tilde{x}}_{k},{\tilde{y}}_{k}\right)=\left(x_{i}\pm \Delta x_{k},x_{i}\pm \Delta y_{k}\right) \end{array} \end{array}\right.$$

where A and V denote the numbers of real and virtual anchors, respectively.
**Algorithm 1.** Generator for the coordinates of the virtual anchorsInput: BorderLength, NodeAmount, BeaconAmount, Span, V;  C (generation of coordinates of all nodes);  Beacon = [C(1,1:BeaconAmount);C(2,1:BeaconAmount)]; % Coordinates of real anchors  Output:    1.   for V = 5:5:25    2.            k = BeaconAmount × V;    3.            for i = NodeAmount:−1:BeaconAmount +1;    4.                     C(:,i + k) = C(:,i); % Shift unknown nodes to leave room for virtual ones    5.            end    6.            n = 1;    7.            for i = 1:V + 1:k + BeaconAmount    8.                     Vcx = Span × randn(1,V);    9.                     Vcy = Span × randn(1,V);    10.                    for j = 0:V    11.                             if (j = 0)    12.                                         C1(:,i + j) = Beacon(:,n);    13.                             else    14.                                         C(1,i + j) = C(1,i) + Vcx(1,j);    15.                                         C(2,i + j) = C(2,i) + Vcy(1,j);    16.                                         *Bind* C(:,i+j) *within* (BorderLength)^2^
*square if outside*
   17.                             end    18.                    end    19.                    n = n + 1;    20.           end    21.           n = n − 1; % Total number of real and virtual anchors n = BeaconAmount × (V + 1)

**Figure 3:**
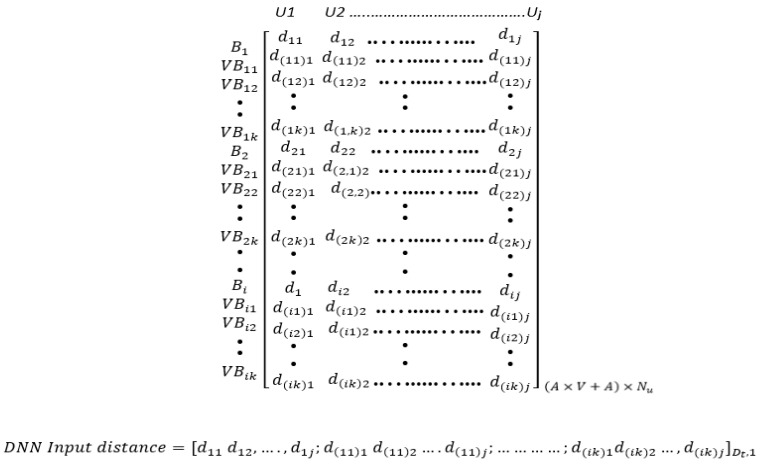
The augmented input data format.

This augmentation strategy involving virtual anchors significantly expands the dataset, providing a more extensive and diverse set of training instances for the proposed DNN model. The input data for the proposed DNN framework is the distance between the real anchors and their virtual anchors, and the unknown nodes. The data are then structured or shaped to be in a format suitable for training, often represented in matrix form. The format of the input data for the DNN is a single array form, as depicted in Figure 3.

In Figure 3, $B_{i}$ and ${\mathrm{VB}}_{\mathrm{ik}}$ represent the locations of the real anchors and virtual anchors, respectively, where (i = 1, …, A), A being the total number of real anchors, and (k = 1, …, V), and V being the total number of virtual anchors. U_j_ denotes the unknown nodes, where (j = 1, …, $N_{u}$ ), with $N_{u}$ being the total number of unknown nodes. The distance between (the real anchors and their virtual anchors), and the unknown nodes, is denoted as $d_{\left(\mathrm{ik}\right)j}$ . The training data size ( $D_{t}$ ) is described in Equation (8):

(8) $${\;D}_{t}=\left(A\times V+A\right)\times N_{u}.$$

**Figure 4:**
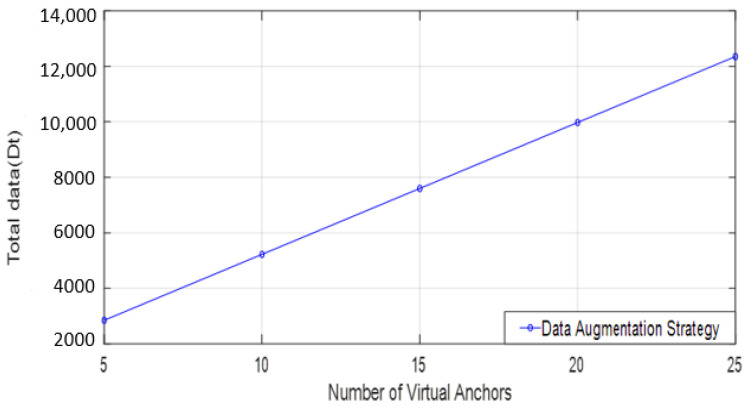
Total data size of virtual augmentation strategy.

In this scenario, the dataset composition is determined by the number of real anchors (A = 5), unknown nodes ( $N_{u}$ = 95), and the presence or absence of virtual anchors (V = 0), the total data size ( $D_{t}$ ) is calculated as 475 using the formula of Equation (7). However, introducing virtual anchors, as exemplified with V = 20, leads to a substantial increase in the total data size, reaching up to 9975, as shown in Table 1 and Figure 4.

## 4. DNN-Based Estimated Distance Correction

To obtain a deeper understanding of DNNs, it is necessary to revisit the basics of ANNs. ANNs have been used in several areas, such as engineering applications and WSN applications [14,19]. Several types of neural networks are described in [13]. Generally, an ANN can be defined as a system or mathematical model that consists of many nonlinear artificial neurons running in parallel and may be generated as one layered or multilayered. An ANN consists of a network of neurons organized in input layers, output layers and hidden layers. Different types of networks can be implemented by varying the structure of the weights and the activation functions of the neurons. Neural network systems can learn how to approximate relationships between inputs and outputs without being overcome by the complexity and size of the problem. The training of ANN using the backpropagation (BP) technique typically occurs in three main steps: the feedforward of input training, the backpropagation of the error, and the update of weights and biases.

**Figure 5:**
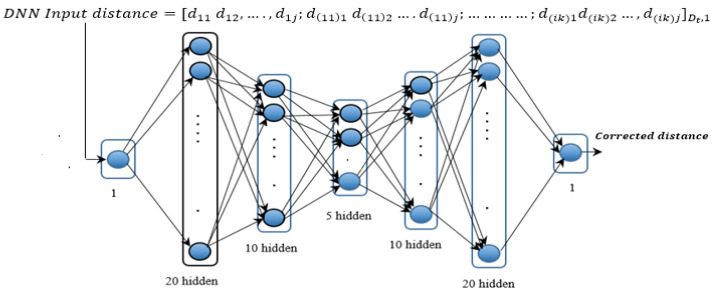
Proposed DNN framework.

ANNs, as highly efficient computational methods, find widespread applications in knowledge representation, machine learning, and predicting output responses in complex systems [20]. Recent advancements have underscored their effectiveness and led to notable achievements in diverse domains [21]. In the domain of ANN training, various processes have been employed [22]. This method is characterized by two essential stages: forward propagation, and backward propagation [23]. Localization system based on WSN and backpropagation-based BP-ANN have been practically implemented to detect and determine the position of an Alzheimer’s patient in an indoor environment [14]. To achieve a minimal localization error, a thorough exploration of various DNN architectures was undertaken, considering different combinations of hidden layers and neurons. Through this iterative process, an optimal DNN architecture emerged, characterized by one input layer (referred to as DNN input distance), five hidden layers with neuron counts of (20, 10, 5, 10, and 20,) and a single output layer (referred to as corrected distance), as depicted in Figure 5.

The process of localization accuracy involved the meticulous collection of DNN data input for the purpose of training, testing, and validation. The dataset was judiciously partitioned, allocating 70% for training, 15% for testing, and an additional 15% for validation. The iterative process for training the DNN was extended up to 1000 iterations, a crucial step undertaken to enable the DNN to reach an optimal normalized root-mean-square error (NRMSE).

## 5. Simulation and Performance Analysis

**Figure 6:**
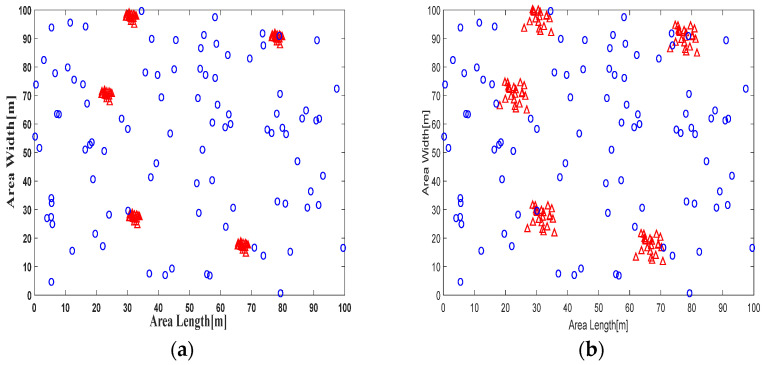
Sensor network configuration 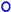: unknown nodes 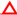: real and virtual anchors (**a**,**b**).

The experimental region was defined by the parameters outlined in Table 2; the nodes were randomly deployed. We also carried out a series of experiments. This section presents one case as an example of node deployment under the influence of network settings, specifically by changing the amount of virtual anchor and span ranges, as illustrated in Figure 6a. This figure provides a visualization of unknown nodes enclosed by 5 real anchors, each surrounded by 20 virtual anchors, with a span equal to 1 m. Figure 6b showcases another scenario where unknown nodes and 5 real anchors are surrounded by 20 virtual anchors around each real anchor, but with a span equal to 6 m. These visual representations offer a concrete example of node deployment configurations, demonstrating the impact of the amount of virtual anchor, and varying span ranges in the simulation setup.

### 5.1. Experiment Results

To verify the performance of the proposed Dv-hop + DNN correction algorithm, the simulations were separately carried out on the Dv-hop algorithm and the improved Dv-hop + DNN correction algorithm across various values of spans and node communication ranges within a randomly selected square area. The evaluation metric employed for this comparison was the normalized root-mean-square error (NRMSE), calculated using Equation (9):

(9) $$\mathrm{NRMSE}=\left(\sum_{i=0}^{U}\sqrt{{\left(x_{i}-{\hat{x}}_{j}\right)}^{2}+{\left(y_{i}-{\hat{y}}_{j}\right)}^{2}}/\left(N_{u}\times R\right)\right)$$

where $\left(x_{i},y_{i}\right)$ denotes the real position of the unknown node, and $\left({\hat{x}}_{j},{\hat{y}}_{j}\right)$ represents the estimated position (Dv-hop) or corrected position (Dv-hop + DNN correction) of the unknown node. The remaining parameters are defined in Table 1.

**Figure 7:**
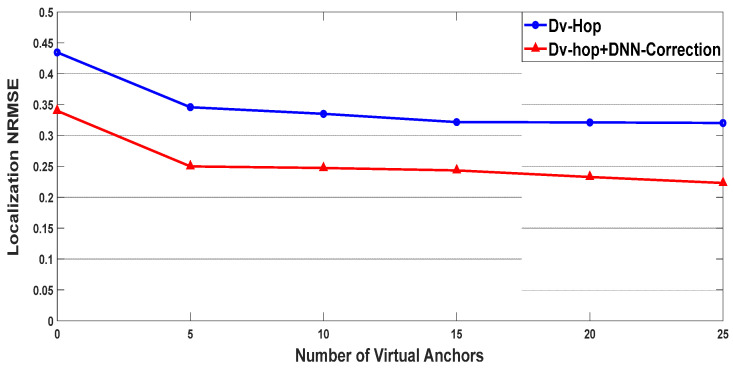
NRMSE performance with different virtual anchors.

**Figure 8:**
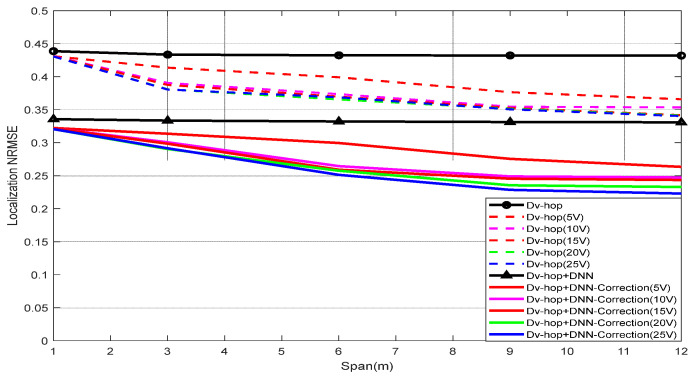
NRMSE performance versus span values.

**Figure 9:**
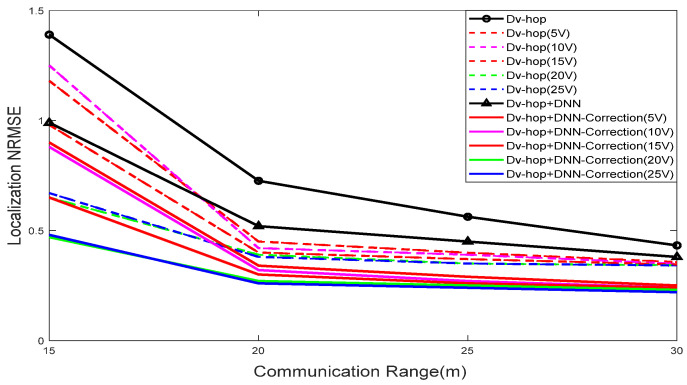
NRMSE performance versus communication range.

The comparison of the localization NRMSE for two different algorithms, Dv-hop and a proposed DNN-correction algorithm, are presented in Figure 7, and a comparison was made of various values of virtual anchors, in Figure 8 concerning span, and in Figure 9 with changes in the communication range. These figures illustrate that the accuracies of all algorithms improved, as expected, with an increasing span and communication range. However, the proposed approach consistently outperformed the Dv-hop algo-rithm in terms of localization accuracy. The error in localizing unknown nodes de-creased with a higher number of virtual anchor nodes in the proposed approach. The positioning errors were smaller with the proposed approach compared to the Dv-hop algorithm, even when the number of virtual anchor nodes was the same. Gradually changing the number of virtual anchor nodes yielded different results in various sce-narios. The proposed approach demonstrates increasing superiority over the Dv-hop approach as the number of virtual anchor nodes rises. Importantly, under the same conditions, the NRMSE of the proposed approach with an augmentation system was smaller than that of the Dv-hop algorithm.

**Figure 10:**
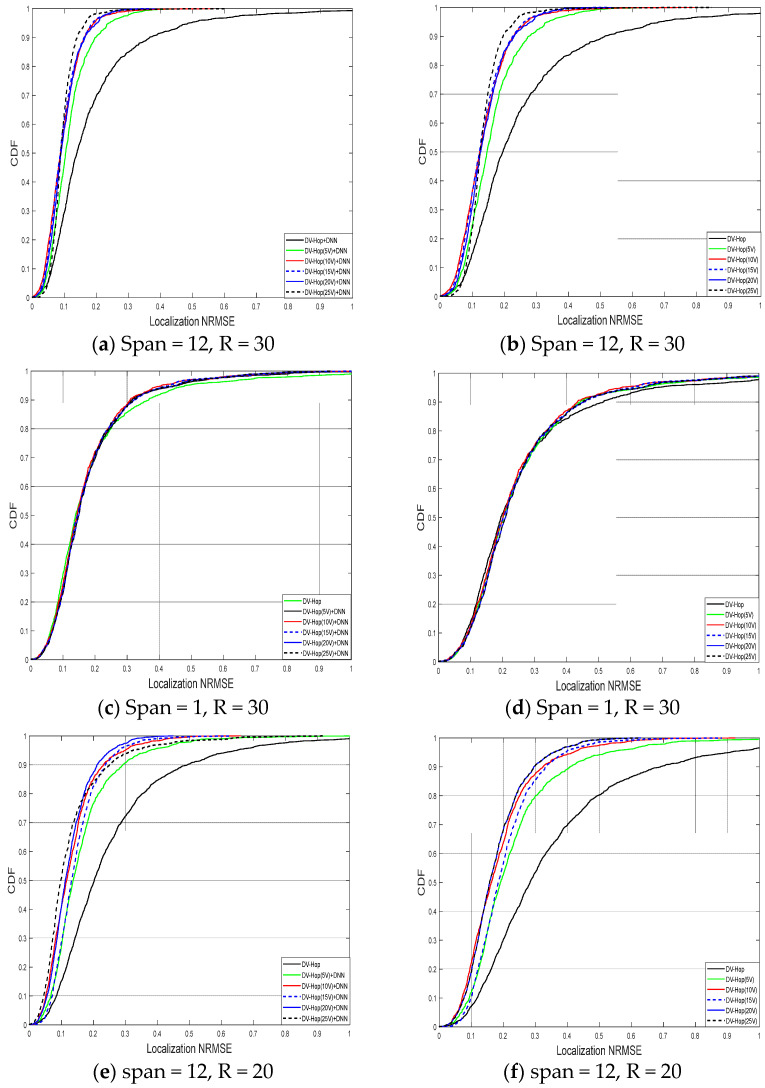
NRMSE CDFs for Dv-hop + DNN in (**a**,**c**,**e**) and Dv-hop in (**b**,**d**,**f**).

Figure 10 illustrates the cumulative distribution function (CDF) of the localization NRMSE with experiment parameters, as shown in Table 2. From Figure 10a, using the proposed algorithm Dv-hop + DNNs correction without augmentation system 70%, and with Dv-hop + DNNs correction for (5 V, 10 V, 15 V, 20 V, and 25 V), 90%, 94%, 95%, 96%, and 98%, respectively, of the sensors could estimate their position with an NRMSE less than 0.2. In contrast, from Figure 10b with Dv-hop for (5 V, 10 V, 15 V, 20 V, and 25 virtual), 76%, 82%, 82%, and 91%, respectively, of the sensors could estimate their position, and only about 50% with Dv-hop without adding virtual anchors. This proves even more the accuracy of the proposed localization algorithm. Figure 10c shows when the span is very small (without and with 5 to 25 virtual), using the Dv-hop + DNN correction algorithm, 71% of nodes estimate their position within a NRMSE value of less than 0. 2, and no more than 50% using Dv-hop (without and with 5 to 25 virtual), as shown in Figure 10d. Meanwhile, Figure 10e and Figure 10f show the effect of communication range when reduced from 30 m to 20 m, for two case Dv-hop and Dv hop + DNNs. The Dv-hop + DNNs correction without augmentation system 50%, and with Dv-hop + DNNs correction for (5 V, 10 V, 15 V, 20 V, and 25 V), 78%, 81%, 82%, 86%, and 84%, respectively, of the sensors could estimate their position with an NRMSE less than 0.2. In contrast, from Figure 10f with Dv-hop for (5 V, 10 V, 15 V, 20 V, and 25 V), 55%, 62%, 69%, and 67% of the nodes achieve the same accuracy with and only about 50% with Dv-hop without adding virtual anchors. From the simulations, we observed that the proposed approach kept improving noticeably with a larger number of virtual anchor nodes up to 20 when the total training data size was 9975, as shown in Table 1. Performance gains started to saturate beyond that threshold.

These further prove the efficiency of the Dv-hop + DNN algorithm, and that the DAS has a sufficient effect for correcting the location of coordinates of unknown nodes.

#### 5.1.1. Effect of Span

The evaluation of the Dv-hop and Dv-hop + DNN correction algorithms was conducted with systematic adjustments made to the span (distance between real anchors and virtual anchors) at values of 1 m, 3 m, 6 m, 9 m, and 12 m. The foundational aspects of the WSN model, detailed in Table 2, remained unchanged throughout these experiments. The experimental outcomes, detailed in Table 3 and Table 4, highlight the performance of the Dv-hop, Dv-hop + virtual, and Dv-hop + DNN correction algorithms under varying span values, assessed through NRMSE values. Remarkably, the NRMSE obtained by the proposed Dv-hop + DNN correction consistently ranked first, particularly when virtual anchors were strategically introduced around the real anchors. This observation underscores the algorithm’s superior performance in terms of both accuracy and cost efficiency.

#### 5.1.2. Effect of Node Communication Range

To test the Dv-hop and Dv-hop + DNN correction algorithms under the effect of communication range. The communication range (R) of the nodes was systematically varied across different values, 15 m, 20 m, 25 m, and 30 m, while keeping the rest of the WSN model unchanged. The experimental results, presented in Table 5 and Table 6, illustrate the performance of the algorithms under these varying communication ranges, with the evaluation based on NRMSE values. Lower NRMSE values indicate higher accuracy in node localization. Generally, an increase in the communication radius corresponds to a decrease in NRMSE values, signifying improved accuracy. It is noteworthy that indiscriminate expansion of the communication range may not always be the optimal strategy. While a larger communication range enhances the accuracy of hop counts between sensor nodes, leading to better connectivity, the optimal approach is contingent on the specific requirements of the WSN. A larger communication range provides sensor nodes with more neighbors, facilitating more accurate hop counts and enhanced connectivity, enabling each unknown node to leverage a greater number of anchors for improved self-localization.

### 5.2. Performance Analysis

This section explores the advantages of the proposed Dv-hop + DDNs correction compared to other state-of-the-art optimization algorithms, including those employed by EFPA-G [24] and WRCDv-hop [25]. Many contemporary optimization algorithms, such as those utilized by EFPA-G and WRCDv-hop, leverage intelligent optimizers to estimate the locations of unknown nodes. The complexity of these algorithms depends on various factors, including the number of real anchors and communication range, leading to increased accuracy at the expense of higher energy consumption. However, the use of many real anchors can be cost-prohibitive and may pose challenges in terms of training data size. To address this issue, we introduced a DAS that virtually increases the number of anchors, mitigating the prohibitive cost associated with the deployment of many real anchors.

## 6. Conclusions

This article introduced an innovative and precise machine learning-based approach for range-free localization in WSN applications within the IoT. Our methodology presents a cost-effective distance estimation strategy through the development of DNN. The aim is to reduce localization errors and enhance accuracy without incurring additional hardware costs. Additionally, we proposed a DAS that virtually increases the number of anchors, significantly augmenting the training data and leading to more accurate localization. Simulation results illustrate the effectiveness of our DAS in range-free localization for WSNs, particularly with a limited number of real anchors. Notably, our proposed Dv-hop + DNNs correction surpasses the traditional Dv-hop algorithm, demonstrating superior localization accuracy.

## Figures and Tables

**Table 1:** Training data size.

Virtual Anchors	Anchors with Augmentation	$\mathrm{Total}\;\mathrm{Data}\;(D_{t})$
5	30	2850
10	55	5225
15	80	7600
20	105	9975
25	130	12,350

**Table 2:** Experimental parameter.

	Contents of Experiments	
$N_{u}$	Number of unknown nodes	95
*A*	Number of real anchors	5
*V*	Number of virtual anchors	5, 10, 15, 20, 25
*S_a_*	Square area	100 × 100 m^2^
*R*	Communications range	15 m, 20 m, 25 m, 30 m
*σ*	Node density	0.01
*S*	Span	1 m, 3 m, 6 m, 9 m, 12 m

**Table 3:** Result of different values of span for Dv-hop, R = 30 m.

	Span	1 m	3 m	6 m	9 m	12 m
	Dv-hop	43.87%	43.34%	43.24%	43.22%	43.20%
	Dv-hop (5 V)	43.09%	41.37%	39.91%	37.65%	36.58%
	Dv-hop (10 V)	43.09%	39.05%	37.35%	35.45%	35.35%
NRMSE of	Dv-hop (15 V)	43.07%	38.75%	36.97%	35.26%	34.17%
	Dv-hop (20 V)	43.06%	38.08%	36.55%	35.14%	34.11%
	Dv-hop (25 V)	43.06%	38.05%	36.87%	35.05%	34.02%

**Table 4:** Results of different values of span for Dv-hop + DNNs correction, R = 30 m.

	Span	1 m	3 m	6 m	9 m	12 m
	Dv-hop + DNN	33.65%	33.05%	33.04%	33.04%	33.05%
	Dv-hop + DNN (5 V)	32.23%	31.35%	29.95%	27.55%	26.34%
NRMSE of	Dv-hop + DNN (10 V)	32.15%	30.05%	26.45%	24.89%	24.73%
	Dv-hop + DNN (15 V)	32.15%	29.85%	25.91%	24.54%	24.34%
	Dv-hop + DNN (20 V)	32.05%	29.05%	25.76%	23.55%	23.29%
	Dv-hop + DNN (25 V)	32.05%	29.15%	25.12%	22.85%	22.30%

**Table 5:** Result of different values of communication range for Dv-hop (span = 1).

	R	15 m	20 m	25 m	30 m
	Dv-hop	139.89%	72.56%	56.34%	43.22%
	Dv-hop (5 V)	118.45%	45.45%	39.45%	35.58%
NRMSE of	Dv-hop (10 V)	115.67%	42.34%	37.87%	34.35%
	Dv-hop (15 V)	98.87%	40.65%	36.23%	34.17%
	Dv-hop (20 V)	65.82%	39.54%	35.05%	34.11%
	Dv-hop (25 V)	67.72%	38.67%	35.12%	34.02%

**Table 6:** Result of different values of communication range for DNNs (span = 12).

	R	15 m	20 m	25 m	30 m
	Dv-hop + DNN	99.02%	52.55%	45.53%	34.05%
	Dv-hop + DNN (5 V)	90.12%	34.87%	29.34%	25.34%
NRMSE of	Dv-hop + DNN (10 V)	88.98%	32.56%	27.43%	24.73%
	Dv-hop + DNN (15 V)	65.44%	30.33%	26.82%	24.34%
	Dv-hop + DNN (20 V)	47.34%	24.36%	24.25%	23.49%
	Dv-hop + DNN (25 V)	48.54%	25.5%	23.56%	22.60%

## Data Availability

Data sharing is not applicable to this article.

## References

[1] Nguyen D.C., Ding M., Pathirana P.N., Seneviratne A., Li J., Niyato D., Dobre O., Poor H.V. (2022). 6G Internet of things: A comprehensive survey. IEEE Internet Things J..

[2] Paul A.K., Sato T. (2017). Localization in wireless sensor networks: A survey on algorithms, measurement techniques, applications, and challenges. J. Sens. Actuator Netw..

[3] Bianchi V., Ciampolini P., Munari I.D. (2019). RSSI-based indoor localization and identification for Zigbee wireless sensor networks in smart homes. IEEE Trans. Instrum. Meas..

[4] Sundhari R.M., Jaikumar K. (2020). IoT assisted hierarchical computation strategic making (HCSM) and dynamic stochastic optimization technique (DSOT) for energy optimization in wireless sensor networks for smart city monitoring. Comput. Commun..

[5] Niculescu D., Nath B. Ad hoc positioning system (APS) using AOA. Proceedings of the IEEE INFOCOM 2003. Twenty-second Annual Joint Conference of the IEEE Computer and Communications Societies (IEEE Cat. No.03CH37428).

[6] Kumar P., Reddy L., Varma S. Distance measurement and error estimation scheme for RSSI based localization in wireless sensor networks. Proceedings of the 2009 Fifth International Conference on Wireless Communication and Sensor Networks (WCSN).

[7] Voltz P.J., Hernandez D. Maximum likelihood time of arrival estimation for real-time physical location tracking of 802.11 a/g mobile stations in indoor environments. Proceedings of the Position Location and Navigation Symposium.

[8] Wang Y., Wang X., Wang D., Agrawal D.P. (2009). Range-free localization using expected hop progress in wireless sensor networks. IEEE Trans. Parallel Distrib. Syst..

[9] Boukerche A., Oliveira H.A.B.F., Nakamura E.F., Loureiro A.A.F. (2007). Localization systems for wireless sensor networks. IEEE Wirel. Commun..

[10] Niculescu D., Nath B. Ad hoc positioning system (APS). Proceedings of the GLOBECOM’01, IEEE Global Telecommunications Conference (Cat. No.01CH37270).

[11] Abu Alsheikh M., Lin S., Niyato D., Tan H.-P. (2014). Machine learning in wireless sensor networks: Algorithms, strategies, and applications. IEEE Commun. Surv. Tutor..

[12] Bhatti G. (2018). Machine learning based localization in large-scale wireless sensor networks. Sensors.

[13] Chen A.C.H., Jia W.K., Hwang F.J., Liu G., Song F., Pu L. (2022). Machine learning and deep learning methods for wireless network applications. EURASIP J. Wirel. Commun. Netw..

[14] Zainab M., Sadik K.G., Ammar H.M., Ali A.J. (2020). Neural network-based Alzheimer’s patient localization for wireless sensor network in an indoor environment. IEEE Access.

[15] Baird H.S. (1995). Document Image Analysis. Chapter Document Image Defect Models.

[16] Lin C., Weidong S. Virtual big data for GAN based data augmentation. Proceedings of the 2019 IEEE International Conference on Big Data.

[17] Zhang B., Lin J., Du L., Zhang L. (2023). Harnessing data augmentation and normalization preprocessing to improve the performance of chemical reaction predictions of data-driven mode. Polymers.

[18] Wu X., Zhang Y., Yu J., Chang C., Qiao H., Wu Y., Wang X., Eu Z., Duan H. (2022). Virtual data augmentation method for reaction prediction. Sci. Rep..

[19] Paul A., Prasad A., Kumar A. (2022). Review on artificial neural network and its application in the field of engineering. J. Mech. Eng. PRAKASH.

[20] Amirsadri S., Mousavirad S.J., Ebrahimpour-Komleh H. (2018). A Levy_fight-based grey wolf optimizer combined with back-propagation algorithm for neural network training. Neural Comput. Appl..

[21] Yang C., Kim H., Adhikari S.P., Chua L.O. (2017). A Circuit-based neural network with hybrid learning of backpropagation and random weight change algorithms. Sensors.

[22] Tarigan J., Diedan R., Suryana Y. (2017). Plate recognition using backpropagation neural network and genetic algorithm. Procedia Comput. Sci..

[23] Alaa A.H., Bekir K., Mohammad S.S. (2016). Back-propagation algorithm with variable adaptive momentum. Knowl. Based Syst..

[24] Jun Z., Ting Y., Wenwu X., Zhihe Y., Dan Y. (2023). An enhanced flower pollination algorithm with gaussian perturbation for node location of a WSN. Sensors.

[25] Cui H., Wang S., Zhou C. (2023). A high-accuracy and low-energy range-free localization algorithm for wireless sensor networks. EURASIP J. Wirel. Commun. Netw..

